# Deficiency of apoA-IV in Female 129X1/SvJ Mice Leads to Diet-Induced Obesity, Insulin Resistance, and Decreased Energy Expenditure

**DOI:** 10.3390/nu15214655

**Published:** 2023-11-02

**Authors:** Jie Qu, Dong Wu, Chih-Wei Ko, Qi Zhu, Min Liu, Patrick Tso

**Affiliations:** 1 Medpace Reference Laboratories, LLC, 5365 Medpace Way, Cincinnati, OH 45227, USA; j.qu@medpace.com; 2 Department of General Surgery, Qilu Hospital of Shandong University, Jinan 250012, China; wudongsdu@hotmail.com; 3 Chroma Medicine, 201 Brookline Ave, Suite 1101, Boston, MA 02215, USA; ckbio87@gmail.com; 4 Department of Pathology and Laboratory Medicine, Metabolic Diseases Institute, University of Cincinnati, 2180 E Galbraith Road, Cincinnati, OH 45237, USA; zhuqu@ucmail.uc.edu (Q.Z.); lium@ucmail.uc.edu (M.L.)

**Keywords:** apolipoprotein A-IV, 129X1/SvJ mice, obesity

## Abstract

Obesity is one of the main risk factors for cardiovascular diseases, type II diabetes, hypertension, and certain cancers. Obesity in women at the reproductive stage adversely affects contraception, fertility, maternal well-being, and the health of their offspring. Being a major protein component in chylomicrons and high-density lipoproteins, apolipoprotein A-IV (apoA-IV) is involved in lipid metabolism, food intake, glucose homeostasis, prevention against atherosclerosis, and platelet aggregation. The goal of the present study is to determine the impact of apoA-IV deficiency on metabolic functions in 129X1/SvJ female mouse strain. After chronic high-fat diet feeding, apoA-IV^−/−^ mice gained more weight with a higher fat percentage than wild-type (WT) mice, as determined by measuring their body composition. Increased adiposity and adipose cell size were also observed with a microscope, particularly in periovarian fat pads. Based on plasma lipid and adipokine assays, we found that obesity in apoA-IV^−/−^ mice was not associated with hyperlipidemia but with higher leptin levels. Compared to WT mice, apoA-IV deficiency displayed glucose intolerance and elevated insulin levels, according to the data of the glucose tolerance test, and increased HOMA-IR values at fasting, suggesting possible insulin resistance. Lastly, we found obesity in apoA-IV^−/−^ mice resulting from reduced energy expenditure but not food intake. Together, we established a novel and excellent female mouse model for future mechanistic study of obesity and its associated comorbidities.

## 1. Introduction

Obesity is one of the high-risk factors for type II diabetes, cardiovascular disease, hypertension, and certain cancers, which has become a new worldwide epidemic [1,2]. Moreover, obesity in women at the reproductive stage adversely affects fertility, increases maternal morbidity and mortality, and causes lasting effects on their children [3,4]. Therefore, it is important to determine factors that affect susceptibility to obesity and understand the underlying mechanism in developing new approaches to treat obesity and metabolic disorders. To achieve this goal, it has become imperative to develop animal models mimicking the characteristics of human obesity and its co-morbidities. Contrary to numerous male mouse models that developed obesity, fewer female models have been established for obesity studies and obesity-related disorders partly due to the protective role of the gonadal hormone, estradiol, in females with respect to gaining excessive visceral fat [5]. So far, obesity has been reported in female mice with mutations in melanocortin receptors [6,7,8,9], leptin receptor [10], estrogen receptor [11,12], and a few others. Furthermore, a handful of them have developed obesity when treated with a high-fat diet, the most popular cause of obesity in humans. Here, we aim to identify an additional female mouse model showing diet-induced obesity.

Apolipoprotein A-IV (ApoA-IV) is an apolipoprotein primarily produced by the small intestinal enterocytes upon lipid absorption and secreted into intestinal lymph associated with chylomicrons [13,14]. In plasma, chylomicrons are metabolized by lipoprotein lipase, and triglycerides are extracted by tissues. Due to the shrinking in the surface area, apoA-IV detaches from the chylomicron particle to be associated with HDL or as circulating free apoA-IV [15,16]. ApoA-IV exerts a major effect on lipid absorption and metabolism [17]. In addition, it acts as a satiety factor, promotes reverse cholesterol transport, confers anti-oxidant and anti-inflammatory activities, and prevents platelet aggregation [18]. More recently, studies using apoA-IV^−/−^ mice on C57/BJ6 background have revealed its function in improving glucose homeostasis by enhancing insulin secretion [19]. It also inhibits hepatic gluconeogenesis through interaction with nuclear receptors, NR1D1 and NR4A1 [20].

Given that apoA-IV^−/−^ mice were originally generated on a mixed background of C57BL/6J and 129X1/SvJ mouse strains [21], our lab first backcrossed the original apoA-IV^−/−^ mice onto a C57BL/6J mouse line because of the C57BL/6J background being commonly used for cardiovascular research. In our previous studies, we demonstrated that apoA-IV^−/−^ mice were deficient in regulating glucose homeostasis [19]. However, comparable body weight, food intake, and lipid metabolism were observed between male apoA-IV^−/−^ and WT mice on C57BL/6J background. In this study, we backcrossed apoA-IV^−/−^ onto 129X1/SvJ background and found that they developed obesity and insulin resistance under a chronic high-fat diet (HFD). Whereas male 129X1/SvJ apoA-IV^−/−^ mice showed higher susceptibility to obesity, described in detail in a separate manuscript, we were excited to learn that female 129X1/SvJ apoA-IV^−/−^ mice also showed obesity under HFD.

In this study, female apoA-IV^−/−^ mice, backcrossed to 129X1/SvJ background for 8 generations, were placed under a 20% butter fat diet for 16 weeks and characterized. Body weight, body composition, and food intake were monitored weekly, while blood samples after a 5-h fast were collected biweekly. A glucose tolerance test was conducted every 4 weeks, and energy expenditure was performed at 8 and 16 weeks. The levels of lipid profiles (triglycerides, cholesterol, phospholipids, and fatty acids), leptin, and adiponectin were measured at the end of the study. Our data suggest that apoA-IV deficiency in female 129X1/SvJ mice results in obesity, insulin insensitivity, and decreased energy expenditure under 16-week HFD.

## 2. Materials and Methods

### 2.1. Animal Study

Female mice in C57BL/6 and 129X1/SvJ mixed background were originally obtained from Dr. J. L. Breslow’s lab at Rockefeller University [21]. We conducted 8 generations of backcrossing to 129X1/SvJ mice from The Jackson Laboratory (Bar Harbor, ME, USA). Genomic DNA of the 129X1/SvJ apoA-IV^−/−^ mice was extracted, and the absence of apoA-IV was validated via PCR amplification. All mice were bred and housed in the facility of the University of Cincinnati, where they were maintained in a 12:12-h light–dark cycle and at 22 °C with ad libitum access to food and water. The standard chow diet, Teklad 7912 (Teklad Diets, Madison, WI, USA), contains 5.8% fat by weight, providing 17% of total calories (3.1 kcal/g). The HFD, Research Diet D03082706 (Research Diets, New Brunswick, NJ, USA), contains 20% fat by weight, providing 40% of total calories (4.54 kcal/g). In this study, after 12 weeks of being on a chow diet, animals were switched to a HFD feeding for a subsequent 16 weeks. Weekly body weight and food consumption were measured throughout the study. Tail blood was collected biweekly from animals that had been fasted for 5 h. Animals were euthanized at the end of the study, and different fat pads were harvested and weighed. All animal protocols were approved by the University of Cincinnati’s Institutional Animal Care and Use Committee and in compliance with the National Institutes of Health Guide for the Care and Use of Laboratory Animals.

### 2.2. Body Composition

Body composition analysis of aged-matched female apoA-IV^−/−^ and WT mice was determined via an EchoMRI (Magnetic Resonance Imaging)-100 system (Echo Medical System, Houston, TX, USA). This analysis assessed fat, lean, free water, and total water masses throughout the entire body of live rodents. The fat (lean) percentage was calculated by dividing whole body fat (lean) mass by total body weight and expressed as a fraction of 100.

### 2.3. Quantification of Adipocyte Size

Periovarian adipose tissues from both WT and apoA-IV^−/−^ mice were collected and fixed in 10% neutral buffered formalin for 48 h at 4 °C, followed by soaking in 70% ethanol at 4 °C for 24 h. Fixed tissues were sent to the Pathology Research Core at Cincinnati Children’s Hospital (Cincinnati, OH, USA) and processed for paraffin embedding, followed by sectioning and staining with hematoxylin and eosin. Five 20-μm sections from different levels of the tissue from each mouse (at least 250 adipocytes per mouse) were analyzed for adipocyte size with an Olympus BX61 microscope (Center Valley, PA, USA) and the freely available open-access software ImageJ (http://rsbweb.nih.gov/ij/, accessed on 15 April 2018) [22].

### 2.4. Measurement of Leptin and Adiponectin Levels

Plasma leptin and adiponectin levels were measured using Mouse Leptin and Mouse Adiponectin ELISA kits, respectively (EMD Millipore Corporation, Billerica, MA, USA), following the manufacturer’s protocols.

### 2.5. Measurement of Plasma Lipid Contents

The levels of triglyceride (TG), cholesterol, phospholipid, and non-esterified fatty acids (NEFA) were measured in plasma samples from animals that had been fasted for 5 h. TG concentrations were assessed using a Randox triglycerides assay kit (Randox Laboratories Ltd., Kearneysville, WV, USA). Cholesterol levels were quantified via Infinity cholesterol assay kit (Thermo Fisher Scientific, Waltham, MA, USA). Phospholipid and NEFA concentrations were measured with the phospholipids assay kit and the HR series NEFA-HR (2) kit (Wako Life Sciences, Inc., Mountain View, CA, USA), respectively. All measurements were conducted following the manufacturer’s manuals.

### 2.6. Analysis of Indirect Calorimetry and Physical Activity

An open circuit indirect calorimetry system (Columbus Instruments Oxymax system) coupled with OPTO-M3 Sensor system (Columbus Instruments, Columbus, OH, USA) was applied to determine oxygen consumption (O_2_) and carbon dioxide (CO_2_) production in mice, as well as physical activity. In brief, mice were individually housed and allowed 1-day acclimation in metabolic chambers before data collection. All measurements were conducted for 24 h when mice were maintained at a temperature of 25 °C and had free access to water and food. The respiration quotient (RQ) was calculated by dividing the CO_2_ volume produced to O_2_ consumed (VO_2_) by mice. The rate of energy expenditure (heat) was calculated using the following equation: Heat = (3.815 + 1.232 × RQ) × VO_2_ and represented as Cal/h/kg body weight (BW). The physical activity of each mouse was evaluated by placing the metabolic chamber in Smart Frame stainless steel cage rack frames (Hamilton-Kinder, Poway, CA, USA), which utilizes infrared beams to monitor an animal’s movement in the *x*, *y*, and *z* axes. Activity counts (beam interruptions) were recorded every 60 min during both light and dark cycles. The total activity during 24 h in the calorimeter was calculated by adding all the ambulatory counts in the X direction.

### 2.7. Glucose Tolerance Assay

Intraperitoneal glucose tolerance tests (IPGTT) were carried out in 12-week-old mice on a chow diet and those on a high-fat diet for 4, 8, 12, and 16 weeks. The mice were fasted for 5 h, followed by i.p. glucose injection (2 g/kg body weight). Blood glucose levels were measured using a glucometer (Freestyle Lite; Abbott, Chicago, IL, USA), and tail blood was collected before, and at 15, 30, 60, and 120 min after glucose injection. Plasma insulin levels were assessed using a Rat/Mouse insulin ELISA kit (EMD Millipore Corporation, Billerica, MA, USA) based on the manufacturer’s protocol.

### 2.8. Data Analysis

Homeostatic Model Assessment of Insulin Resistance (HOMA-IR) was evaluated for the 129X1/SvJ WT and apoA-IV^−/−^ mice using the following equation [23]:

HOMA-IR = (Fasting glucose levels (mg⁄dl) × fasting insulin levels (mU⁄ L))/405.

Fasting insulin levels measured in this study were in ng/mL unit and were converted to mU/L before HOMA-IR calculation.

### 2.9. Statistical Analysis

All data were displayed as mean ± standard error of the mean (SEM). Graphs were drawn, and statistical analysis was conducted using PRISM 8.0 software (GraphPad Software, La Jolla, CA, USA). Interactions between genotypes (WT vs. apoA-IV^−/−^) were analyzed via regular two-way ANOVA when appropriate. One-way ANOVA and independent *t*-tests were also used when appropriate. *p*-value of less than 0.05 was indicative of statistical significance; the significance level was indicated as * p < 0.05, ** p < 0.01, *** p < 0.001, and **** *p* < 0.0001.

## 3. Results

### 3.1. ApoA-IV^−/−^ Mice Develop Obesity under High Fat Diet

**Figure 1:**
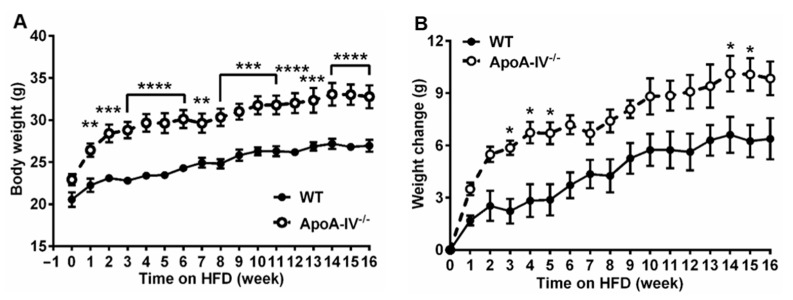
The progression of obesity in apoA-IV^−/−^ mice over a 16-week HFD study. (**A**) Body weight. (**B**) Body weight change. Data are depicted as mean ± SEM (*n* = 6) and analyzed statistically via regular two-way ANOVA. Significant differences relative to the WT group (* p < 0.05; ** p < 0.01; *** p < 0.001; **** p < 0.0001).

Both female wild-type (WT) and apoA-IV^−/−^ mice were fed a chow diet for 12 weeks before feeding on HFD for an additional 16 weeks. When on a chow diet for 12 weeks, apoA-IV^−/−^ mice were slightly heavier than WT mice, though the difference did not reach statistical significance (Figure 1A). During HFD feeding, apoA-IV^−/−^ mice weighed significantly more compared to WT mice (Figure 1A), with an average body weight of 32.8 ± 3.3 g for apoA-IV^−/−^ and 26.9 ± 1.8 g for WT mice at the end of the study. In addition, the gain in body weight was more profound from weeks 3 to 5 and from weeks 14 to 15 in apoA-IV^−/−^ mice (Figure 1B).

**Figure 2:**
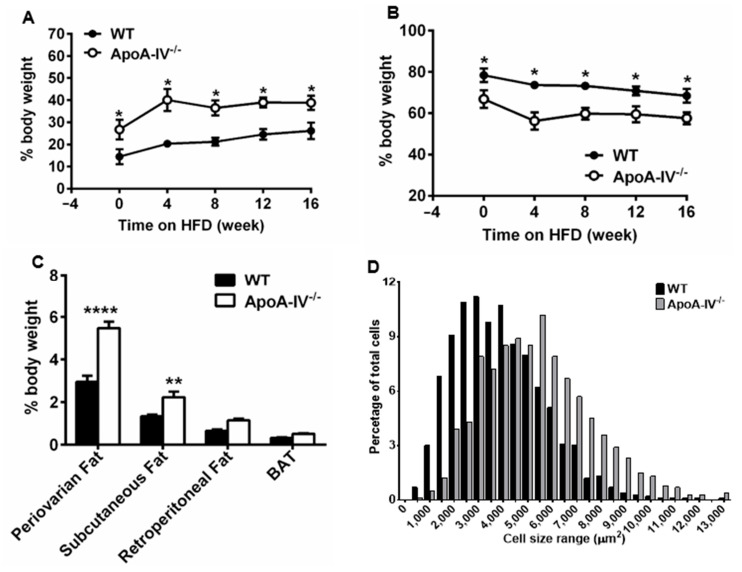
ApoA-IV^−/−^ mice conferred greater adiposity than WT mice. Body composition with the percentage of fat (**A**) and lean (**B**) mass. (**C**) Percentage of periovarian, subcutaneous, and retroperitoneal white adipose tissues (WAT) and brown adipose tissue (BAT) in mice at the end of 16-week HFD study. (**D**) The size distribution of adipocytes in H&E staining of periovarian fat pads collected during animal sacrifice. Data are presented as mean ± SEM (*n* = 6) and analyzed statistically via regular two-way ANOVA. Significant differences relative to the WT group (* p < 0.05; ** p < 0.01; **** p < 0.0001).

To assess the body composition, the nuclear magnetic resonance (NMR)–magnetic resonance imaging (MRI)-based method was used on mice before and at weeks 4, 8, 12, and 16 on HFD. Interestingly, under the chow diet, apoA-IV^−/−^ mice already showed significantly higher fat percentage (26.7 ± 4.4% for mutant mice vs. 14.5 ± 3.4% of body weight for WT mice). After HFD feeding, the fat percentage of apoA-IV^−/−^ mice reached 40.1 ± 5.0% at week 4 and was maintained at a similar level until week 16, whereas the fat percentage steadily increased from 20.3 ± 1.5% to 26.2 ± 3.8% in WT mice. Complementary to fat percentage data, the lean mass percentage of apoA-IV^−/−^ mice exhibited a significantly lower level than WT mice (Figure 2B).

To understand the extent of fat accumulation in different fat tissues, white adipose tissue (periovarian, subcutaneous, retroperitoneal fat pads) and brown adipose tissue were dissected and weighed, and the absolute mass was normalized to body weight. Compared to WT mice, apoA-IV^−/−^ mice had significantly more fat deposited to periovarian and subcutaneous fat pads (Figure 2C). In particular, the absolute weight of periovarian and subcutaneous fat pads of apoA-IV^−/−^ mice was approximately 2.3-fold and 2.1-fold greater than that of WT mice (Supplemental Figure S1 and Table S1). However, no difference was found in retroperitoneal fat and brown adipose tissue between the two groups (Figure 2C). To determine if increased periovarian adiposity resulted from adipocyte hypertrophy, we assessed adipocyte size and revealed that apoA-IV^−/−^ mice contained larger adipocytes than WT mice (Figure 2D). In summary, after HFD feeding, apoA-IV^−/−^ mice were heavier and developed periovarian and subcutaneous adiposity, suggesting they are more susceptible to diet-induced obesity.

### 3.2. ApoA-IV^−/−^ Mice Contain Higher Leptin but Lower Cholesterol and Phospholipid Levels

**Figure 3:**
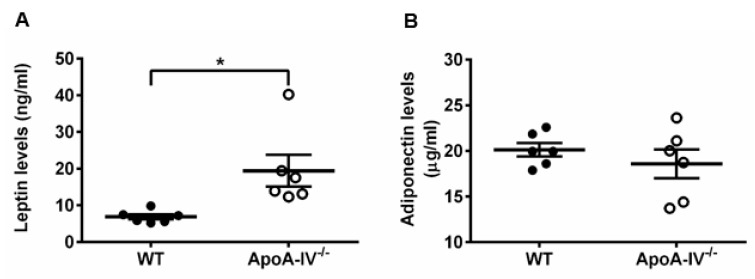
ApoA-IV^−/−^ mice contained higher leptin levels and comparable adiponectin levels than WT mice at the end of the 16-week HFD study. (**A**) Leptin levels. (**B**) Adiponectin levels. Black circles indicate WT mice and white circles indicate ApoA-IV^−/−^ mice. Data are depicted as mean ± SEM (*n* = 6) and analyzed statistically via student *t*-test. Significant differences compared to the WT group (* p < 0.05).

Secreted by adipose tissues, leptin and adiponectin are known to be associated with obesity [24,25]. Therefore, plasma leptin and adiponectin levels were measured via ELISA on Week 16 of HFD feeding. In comparison to WT mice, leptin levels were significantly higher in apoA-IV^−/−^ mice (Figure 3A), consistent with the notion that leptin concentrations are positively correlated with the percentage of body fat [24]. Adiponectin levels, inversely related to obesity [25], were slightly lower in apoA-IV^−/−^ mice (Figure 3B).

**Figure 4:**
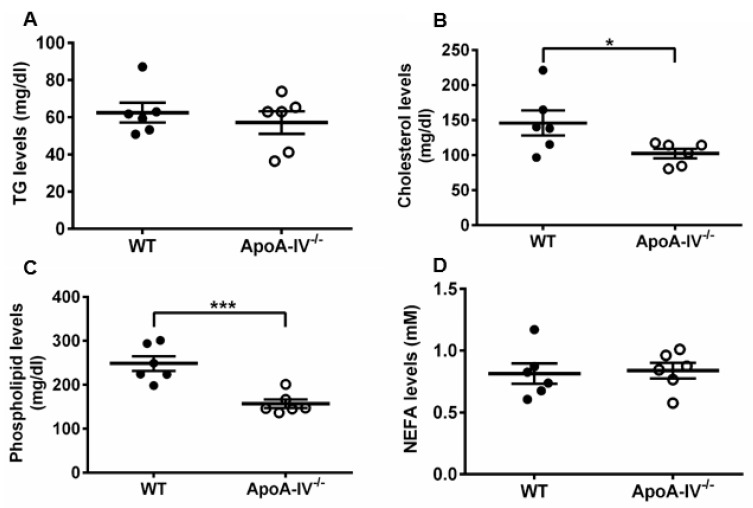
Lipid profile analysis of WT and apoA-IV^−/−^ mice at the end of 16-week HFD study. (**A**) Circulating triglycerides (TG) levels. (**B**) Cholesterol levels. (**C**) Phospholipids levels. (**D**) Non-esterified fatty acid (NEFA) levels. Black circles indicate WT mice and white circle indicate ApoA-IV^−/−^ mice. Data are depicted as mean ± SEM (*n* = 6) and analyzed statistically via student *t*-test. Significant differences relative to the WT group (* p < 0.05; *** *p* < 0.001).

Considering that obesity is often associated with plasma lipid changes, such as increased triglyceride levels [26], the plasma lipid profile was assessed upon completion of the HFD study. TG, cholesterol, phospholipids, and free fatty acid concentrations were determined via enzymatic and colorimetric methods. TG and free fatty acid levels were not significantly different, while cholesterol and phospholipid levels were significantly higher in WT mice than in apoA-IV^−/−^ mice (Figure 4). These data suggest that diet-induced obesity in apoA-IV^−/−^ mice leads to leptin insensitivity and is associated with low cholesterol and phospholipids levels.

### 3.3. Obese ApoA-IV^−/−^ Mice Are Glucose Intolerant and Insulin Resistant

**Figure 5:**
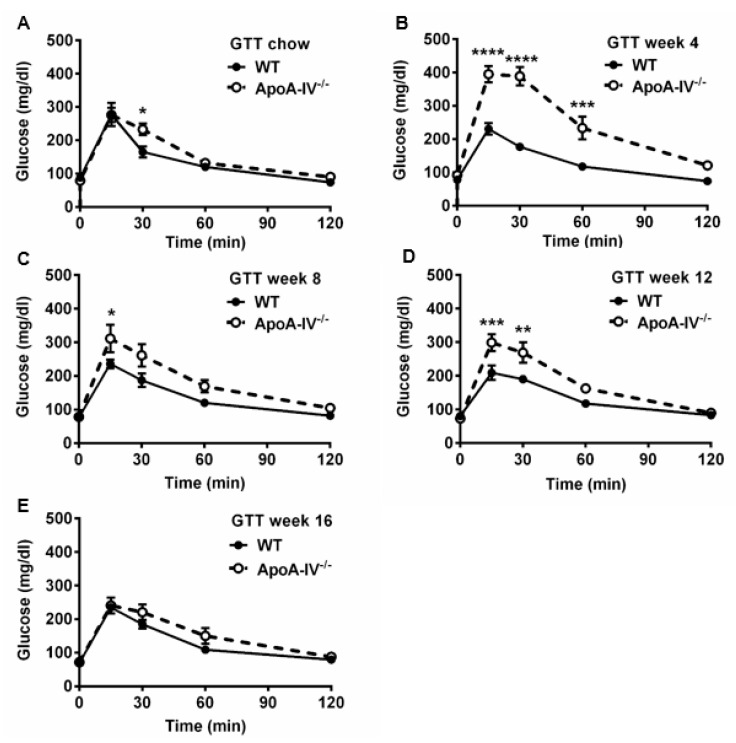
Glucose tolerance test in WT and apoA-IV^−/−^ mice before and during HFD study. Blood glucose levels over 120 min upon glucose challenge at 0 (**A**) and weeks 4 (**B**), 8 (**C**), 12 (**D**), and 16 (**E**) of the HFD study. Data are presented as mean ± SEM (*n* = 6), and statistical analysis of data was conducted via regular two-way ANOVA. Significant differences relative to the WT mice group (* p < 0.05; ** p < 0.01; *** *p* < 0.001; **** *p* < 0.0001).

**Figure 6:**
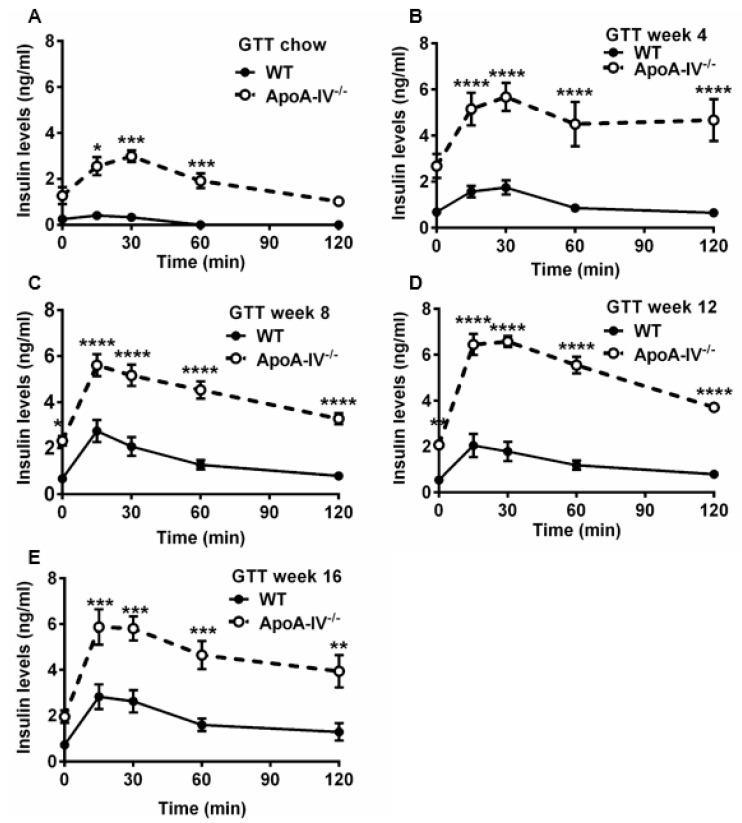
Plasma insulin levels during glucose tolerance test in WT and apoA-IV^−/−^ mice before and during HFD study. Plasma insulin levels throughout 120 min post-post-glucose injection), before (**A**) and on weeks 4 (**B**), 8 (**C**), 12 (**D**), and 16 (**E**) of the HFD study. Data are presented as mean ± SEM (*n* = 6), and statistical analysis of data was conducted via regular two-way ANOVA. Significant differences relative to the WT group (* p < 0.05; ** p < 0.01; *** *p* < 0.001; **** p < 0.0001).

Numerous articles demonstrate that abnormal glucose metabolism is often seen in obese subjects [27]. To understand the ability of glucose handling in both the WT and apoA-IV^−/−^ mice, the glucose tolerance test (GTT) was conducted on mice before and 4, 8, 12, and 16 weeks after feeding HFD. When fed a chow diet, glucose levels in apoA-IV^−/−^ mice peaked at 15 min upon glucose challenge, similar to the levels observed in WT mice; however, they dropped much more slowly than that of WT mice, indicating moderate glucose intolerance in the apoA-IV^−/−^ mice (Figure 5A). After being fed HFD, glucose intolerance became more severe in apoA-IV^−/−^ mice than in WT mice (Figure 5B–D, except at week 16 of the HFD study (Figure 5E). To determine the ability of insulin secretion in response to glucose administration, plasma insulin levels were also measured during GTT via ELISA. Compared to WT mice, apoA-IV^−/−^ mice secreted more insulin to decrease elevated blood glucose concentrations caused by glucose injection before HFD (Figure 6A). Moreover, insulin secretion in apoA-IV^−/−^ mice was enhanced about 2-fold after they were placed on HFD (Figure 6B–E). Together, these findings imply that apoA-IV^−/−^ mice developed insulin resistance over time on HFD.

We noticed that the glucose data observed at 16 weeks (Figure 5E) showed no significant difference in plasma glucose levels between apoA-IV^−/−^ and WT mice. One of the possibilities is a compensatory mutation. The compensatory mutation is when a change in one gene makes things worse but is followed by another change in a gene that helps to fix things [28]. In this specific situation, after chronic HFD consumption, estrogen may trigger another gene(s), e.g., glucagon-like peptide 1 (GLP-1) with overlapping functions of apoA-IV, to compensate for the loss of the apoA-IV gene, leading to increased estrogen protective effects on glucose metabolism and insulin sensitivity in apoA-IV^−/−^ mice. Compensatory mutation is a necessary and poorly understood evolutionary process. It is worth identifying the potential target genes using an RNA sequencing approach in the future.

**Figure 7:**
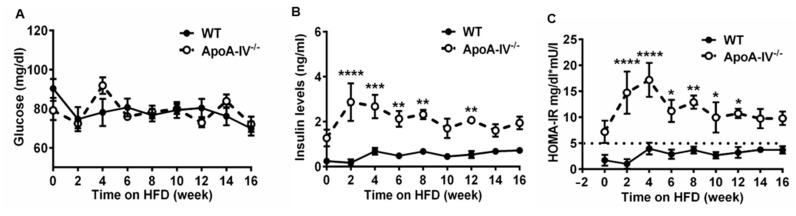
Glucose homeostasis in WT and apoA-IV^−/−^ mice during 16-week HFD study. (**A**) Blood glucose levels. (**B**) Plasma insulin levels. (**C**) HOMA-IR. Data are presented as mean ± SEM (*n* = 6) and analyzed statistically via regular two-way ANOVA. Significant differences relative to the WT group (* *p* < 0.05; ** p < 0.01; *** *p* < 0.001; **** *p* < 0.0001).

To evaluate the degree of insulin resistance during the fasting state, HOMA-IR was employed [23], and values were calculated based on fasting glucose and insulin levels. While no difference in basal plasma glucose levels was seen between the two genotypes, basal insulin levels were much higher in apoA-IV^−/−^ mice at weeks 2, 4, 6, 8, and 12 on HFD (Figure 7A,B). ApoA-IV^−/−^ mice also presented a much higher HOMA-IR index than WT mice, suggesting significant insulin resistance (HOMA-IR > 5) [29].

### 3.4. Energy Expenditure but Not Food Intake Is Reduced in ApoA-IV^−/−^ Mice

**Figure 8:**
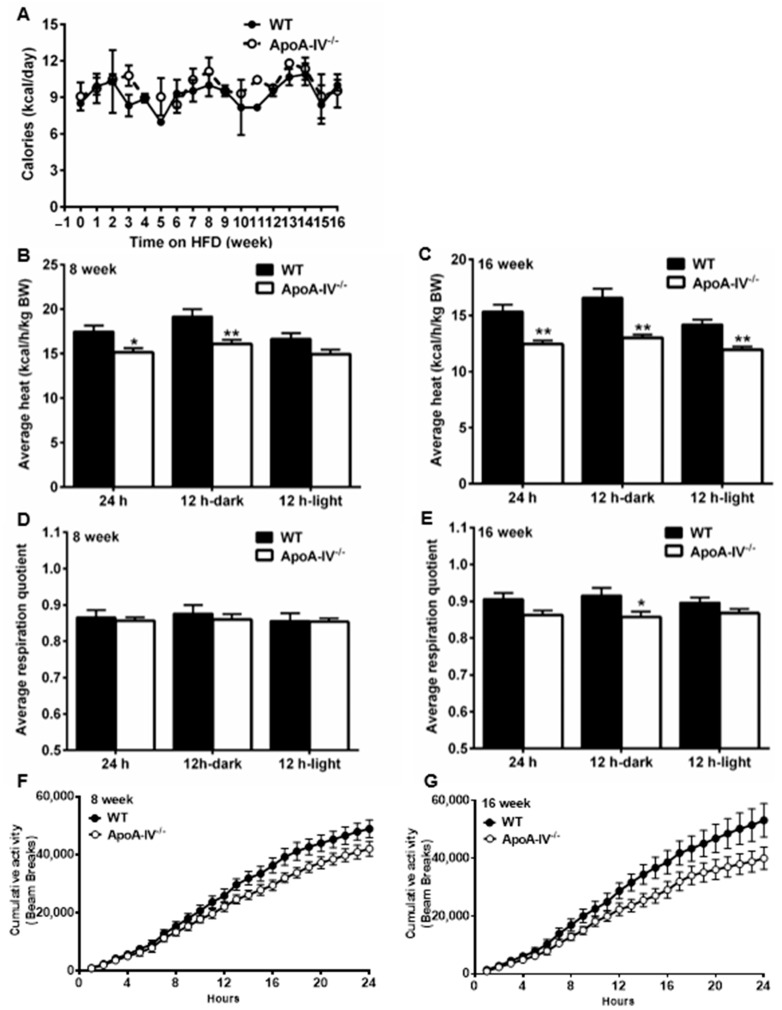
Food intake and energy expenditure of WT and apoA-IV^−/−^ mice during a 16-week HFD study. (**A**) Weekly food intake. Average heat production over 12-h and 24-h time course (**B**,**C**), average respiration quotient over 12-h and 24-h time course (**D**,**E**), and cumulative physical activity over 24-h (**F**,**G**) on Weeks 8 and 16, respectively, after HFD consumption. Data are presented as mean ± SEM (*n* = 6). Data in (**A**,**F**,**G**) were analyzed statistically via regular two-way ANOVA, whereas data analysis in (**B**–**E**) were determined statistically via student *t*-test. Significant differences relative to the WT group (* *p* < 0.05; ** *p* < 0.01).

Obesity is often a result of calorie surplus due to either increased energy intake and/or decreased energy expenditure [30]. To monitor energy intake, mouse daily food intake was tracked every week for 16 weeks. To assess energy expenditure, the indirect calorimetry method was used to calculate respiration quotient (RQ) and energy expenditure rate (heat) on Week 8 and Week 16 of the HFD study. No statistically significant increase in food intake was observed between the two groups (Figure 8A), even though murine models of obesity generally show hyperphagia. On Week 8 and Week 16, O_2_ and CO_2_ consumption were significantly higher in apoA-IV^−/−^ mice than in WT mice in the dark cycle when rodents were more active (Supplemental Figure S2A,B,E,F). On Week 8 and Week 16, total heat production was much lower in apoA-IV^−/−^ than in WT mice (Figure 8B,C). On week 8, this difference was illustrated only in the dark cycle (Figure 8B), whereas the difference on Week 16 was aggravated in both light and dark cycles (Figure 8C). Daily total RQ remains the same on Week 8 and Week 16 (Figure 8D,E). However, RQ levels were lower in apoA-IV^−/−^ mice in the dark on week 16 (Figure 8E). On Week 8 and Week 16, cumulative physical activity was less intense in apoA-IV^−/−^ than in WT mice, though the difference was comparable (Figure 8F,G). Based on these results, obesity in apoA-IV^−/−^ mice results from reduced energy expenditure, presumably heat production, but not increased food intake.

## 4. Discussion

In the present study, we backcrossed apoA-IV^−/−^ mice to 129 X1/SvJ background for eight generations and characterized the differences in metabolic phenotypes between female 129X1/SvJ WT and apoA-IV^−/−^ mice under chronic consumption of a HFD. Our data revealed for the first time that diet-induced obesity occurred in female 129X1/SvJ apoA-IV^−/−^ mice, which also exhibited insulin resistance. Obesity in apoA-IV^−/−^ mice is associated with elevated body fat percentage, likely attributable to adipocyte hypertrophy and an increase in periovarian and subcutaneous adiposity. The adipokine (leptin) secreted from adipocytes was significantly higher in apoA-IV^−/−^ mice than in WT mice. However, compared to WT mice, hyperlipidemia was not observed in apoA-IV^−/−^ mice. Our indirect calorimetry data suggested that normal calorie intake and decreased energy expenditure result in positive energy balance and thus obesity in apoA-IV^−/−^ mice. Obesity-associated insulin resistance was also observed when significantly high insulin levels were required to maintain glycemic control in apoA-IV^−/−^ mice during fasting state and glucose tolerance test.

ApoA-IV^−/−^ mice provide a valuable new model for investigating female obesity and associated health problems. The female sex hormone, estrogen, is known to decrease food intake and promote energy expenditure, thereby preventing obesity [31,32,33]. It was previously demonstrated by Nomelí P. Núñez’s group that female mice are less likely to become obese than male mice [34]. When the ovary was removed from female mice via ovariectomy, they became obese, and the obesity was reversed through the administration of external estrogen [35,36]. Therefore, female obese models are more challenging to establish due to the protective role of estrogen in female animals. Female 129 X1/SvJ apoA-IV^−/−^ mice can be an excellent model for studying female obesity. Previous studies showed that estrogen enhanced central apoA-IV gene expression via estrogen receptor-α, and estrogen’s inhibitory effect on feeding was partially mediated through apoA-IV [37]. Considering that apoA-IV acts downstream of the estrogen pathway, our results imply that the loss of apoA-IV attenuated estrogen’s protective role on weight gain was not unexpected. ApoA-IV^−/−^ mice may offer better opportunities to study obesity and its associated complications than estrogen receptor knockout mice [11,38], in which the physiological effects of estrogen, other than reducing food intake, mainly were compromised.

Compared to WT mice, 129X1/SvJ apoA-IV^−/−^ mice showed reduced energy expenditure, reduced O_2_/CO_2_ consumption, heat production, and general activity, but comparable food intake. These metabolic phenotypes are not unprecedented in apoA-IV global knock-out mice bred on a C57BL/6J background. Dr. Chunmin Lo’s group previously found that male C57BL/6J apoA-IV^−/−^ mice exhibited reduced energy expenditure at multiple times during the dark phase after either one-week or 20-week HFD feeding [39]. In terms of food intake, C57BL/6J WT and apoA-IV^−/−^ mice showed comparable daily food consumption under either short-term or chronic HFD. Under short-term HFD, the hourly average energy expenditure was significantly lower in C57BL/6J apoA-IV^−/−^ mice than in WT mice during the dark phase [39]. Moreover, expressions of key thermogenesis genes *Ucp1*, *Ucp2*, *Cpt1*, and *Ampka1* in BAT were significantly lower in apoA-IV^−/−^ mice under short-term HFD, suggesting that reduced energy expenditure in apoA-IV^−/−^ mice results from decreased UCP-1 dependent BAT thermogenesis [39]. Therefore, BAT thermogenesis may also be impaired in female 129X1/SvJ apoA-IV^−/−^ mice. However, whether *Ucp1* gene expression is reduced in female 129X1/SvJ apoA-IV^−/−^ mice awaits further investigation.

ApoA-IV^−/−^ mice confer insulin resistance, not correlated with higher circulating free fatty acid levels but rather with increased adiposity. A previous study showed that the HOMA-IR index assessed from basal glucose and insulin levels for WT 129X1/SvJ mice was 4.9 ± 0.5, which is considered normal insulin sensitivity. Compared to WT mice, apoA-IV^−/−^ mice had a significantly higher HOMA-IR index (greater than 5) throughout the HFD feeding, indicating that apoA-IV^−/−^ mice exhibit insulin resistance. During the glucose tolerance test, blood glucose and insulin levels were higher in apoA-IV^−/−^ mice than in WT mice at 30 min post glucose injection. In addition, although blood glucose levels were back to normal at 120 min post glucose injection, blood insulin levels remained significantly higher in apoA-IV^−/−^ mice than in WT mice, suggesting insulin resistance in apoA-IV^−/−^ mice in response to glucose excursion. It is widely accepted that insulin resistance is related to extensive lipid deposition in organs [40,41]. When nutrient intake exceeds energy expenditure for a prolonged period, such as chronic consumption of a high-fat diet, excessive energy is stored in adipose tissues, which engenders adipocyte hypertrophy (increase in cell size) and hyperplasia (increase in cell number). The enlargement and lipid overload induce intracellular abnormalities in adipocyte function, particularly endoplasmic reticulum and mitochondria stress [41]. Dysfunction of adipocytes will result in insulin resistance within adipocytes and the production of adipokines, free fatty acids, and inflammatory mediators. Excessive non-esterified fatty acids (NEFA), diacylglycerol, and ceramides secreted to the circulation will deposit and trigger systemic insulin resistance in remote organs, such as the liver, muscle, heart, and vascular beds. Given that circulating NEFA level was comparable between apoA-IV^−/−^ mice and WT mice, insulin resistance in apoA-IV^−/−^ mice may not result from elevated NEFA levels. It is well-established that smaller adipocytes have a strong association with insulin sensitivity [42]. Therefore, we speculate that insulin resistance in apoA-IV^−/−^ mice is attributable to enlarged adipocyte size, a step before the excessive release of NEFA and fatty acid metabolites. Further study is required to decipher how loss of apoA-IV elicits insulin resistance in adipose tissues.

Our data clearly showed that loss of apoA-IV had different metabolic effects on 129X1/SvJ and C57BL/6J mouse strain. 129X1 apoA-IV^−/−^ mice developed obesity and insulin resistance from HFD feeding, whereas C57BL/6J apoA-IV^−/−^ mice showed average body weight and glucose intolerance from insufficient β-cell insulin secretion [19]. We hypothesize that there must be modifying genes or genes that interact with apoA-IV to change its physiological function. These modifying genes are present in different levels between C57BL/6J and the 129X1/SvJ strains. The discovery of the modifying gene(s) will greatly enhance our understanding of the apoA-IV function in rodents and humans. These differences may also be due to genetic variation in apoA-IV levels and variables linked to energy balance and glucose homeostasis between the two strains. The 129 strain was revealed to have approximately 10-fold higher hepatic apoA-IV mRNA levels than the C57 strain, although their intestinal apoA-IV mRNA levels varied by less than 2-fold [43]. ApoA-IV protein structure also exhibited genetic variation between the two strains, with the 129 strain producing the apoA-IV isoform that is one charge more basic than C57 apoA-IV [44]. On the other hand, the 129 strain and C57 strain showed variation in obesity and glucose homeostasis in response to HFD feeding. On an HFD, the 129 strain displayed a greater increase in body weight and glucose intolerance than the C57 strain [45]. Among four commonly used inbred mouse strains (129Xl/SvJ, FVB/N, DBA/2, and C57BL/6), the C57 strain had the highest fasting blood glucose and glucagon, while 129 strain had the lowest blood glucose and insulin [29]. Additionally, in vivo, insulin secretion was greatest in the 129 strain, and response to hyperinsulinemia was intermediate in the C57 strain [29]. Together, these data suggest that apoA-IV in two strains may vary in metabolic functions; hence, deficiency of apoA-IV on two different strains is likely to generate different phenotypic outcomes.

## 5. Conclusions

In this study, we found that apoA-IV^−/−^ female mice gained more weight with a higher fat percentage than WT mice after chronic HFD consumption. These were associated with higher leptin levels but not with hyperlipidemia. Compared to WT mice, apoA-IV deficiency displayed glucose intolerance and elevated insulin levels, suggesting insulin resistance. Additionally, we found obesity of apoA-IV^−/−^ resulted from reduced energy expenditure but not food intake. The metabolic differences observed between C57 and 129X1 apoA-IV^−/−^ mice provide valuable resources with which to probe further the mechanism by which apoA-IV modulates weight gain and glucose homeostasis in response to diet. In humans, apoA-IV polymorphism leads to differences in lipid binding affinity, lipid absorption, chylomicron metabolism, plasma lipoprotein levels, and, ultimately, the heterogeneous response of plasma lipids to diet [46]. The gained knowledge is expected to eventually apply to humans for individualized treatment of obesity and its associated comorbidities, such as diabetes and cardiovascular diseases.

## Data Availability

Not applicable.

## Supplementary Materials

The following supporting information can be downloaded at https://www.mdpi.com/article/10.3390/nu15214655/s1: Figure S1: Representative sections of periovarian fat pads with H&E staining; Figure S2: Indirect calorimetry measurement of WT and apoA-IV^−/−^ mice at week 8 and week 16 of HFD; Table S1. Tissue weight (g) of different fat depots.

## References

[1] Friedrich M.J. (2017). Global Obesity Epidemic Worsening. JAMA.

[2] Dixon J.B. (2010). The Effect of Obesity on Health Outcomes. Mol. Cell. Endocrinol..

[3] Jungheim E.S., Travieso J.L., Hopeman M.M. (2013). Weighing the Impact of Obesity on Female Reproductive Function and Fertility. Nutr. Rev..

[4] Metwally M., Li T.C., Ledger W.L. (2007). The Impact of Obesity on Female Reproductive Function. Obes. Rev..

[5] Stubbins R.E., Holcomb V.B., Hong J., Núñez N.P. (2012). Estrogen Modulates Abdominal Adiposity and Protects Female Mice from Obesity and Impaired Glucose Tolerance. Eur. J. Nutr..

[6] Boston B.A., Cone R.D. (1996). Characterization of Melanocortin Receptor Subtype Expression in Murine Adipose Tissues and in the 3T3-L1 Cell Line. Endocrinology.

[7] Huszar D., Lynch C.A., Fairchild-Huntress V., Dunmore J.H., Fang Q., Berkemeier L.R., Gu W., Kesterson R.A., Boston B.A., Cone R.D. (1997). Targeted Disruption of the Melanocortin-4 Receptor Results in Obesity in Mice. Cell.

[8] Chen A.S., Marsh D.J., Trumbauer M.E., Frazier E.G., Guan X.M., Yu H., Rosenblum C.I., Vongs A., Feng Y., Cao L. (2000). Inactivation of the Mouse Melanocortin-3 Receptor Results in Increased Fat Mass and Reduced Lean Body Mass. Nat. Genet..

[9] Ste Marie L., Miura G.I., Marsh D.J., Yagaloff K., Palmiter R.D. (2000). A Metabolic Defect Promotes Obesity in Mice Lacking Melanocortin-4 Receptors. Proc. Natl. Acad. Sci. USA.

[10] Cohen P., Zhao C., Cai X., Montez J.M., Rohani S.C., Feinstein P., Mombaerts P., Friedman J.M. (2001). Selective Deletion of Leptin Receptor in Neurons Leads to Obesity. J. Clin. Investig..

[11] Heine P.A., Taylor J.A., Iwamoto G.A., Lubahn D.B., Cooke P.S. (2000). Increased Adipose Tissue in Male and Female Estrogen Receptor-Alpha Knockout Mice. Proc. Natl. Acad. Sci. USA.

[12] Manrique C., Lastra G., Habibi J., Mugerfeld I., Garro M., Sowers J.R. (2012). Loss of Estrogen Receptor α Signaling Leads to Insulin Resistance and Obesity in Young and Adult Female Mice. Cardiorenal Med..

[13] Utermann G., Beisiegel U. (1979). Apolipoprotein A-IV: A Protein Occurring in Human Mesenteric Lymph Chylomicrons and Free in Plasma. Isolation and Quantification. Eur. J. Biochem..

[14] Simon T., Cook V.R., Rao A., Weinberg R.B. (2011). Impact of Murine Intestinal Apolipoprotein A-IV Expression on Regional Lipid Absorption, Gene Expression, and Growth. J. Lipid Res..

[15] Green P.H., Glickman R.M., Riley J.W., Quinet E. (1980). Human Apolipoprotein A-IV. Intestinal Origin and Distribution in Plasma. J. Clin. Investig..

[16] Ohta T., Fidge N.H., Nestel P.J. (1985). Studies on the In Vivo and In Vitro Distribution of Apolipoprotein A-IV in Human Plasma and Lymph. J. Clin. Investig..

[17] Qu J., Ko C.-W., Tso P., Bhargava A. (2019). Apolipoprotein A-IV: A Multifunctional Protein Involved in Protection against Atherosclerosis and Diabetes. Cells.

[18] Xu X.R., Wang Y., Adili R., Ju L., Spring C.M., Jin J.W., Yang H., Neves M.A.D., Chen P., Yang Y. (2018). Apolipoprotein A-IV Binds AIIbβ3 Integrin and Inhibits Thrombosis. Nat. Commun..

[19] Wang F., Kohan A.B., Kindel T.L., Corbin K.L., Nunemaker C.S., Obici S., Woods S.C., Davidson W.S., Tso P. (2012). Apolipoprotein A-IV Improves Glucose Homeostasis by Enhancing Insulin Secretion. Proc. Natl. Acad. Sci. USA.

[20] Li X., Xu M., Wang F., Ji Y., DavidsoN W.S., Li Z., Tso P. (2015). Interaction of ApoA-IV with NR4A1 and NR1D1 Represses G6Pase and PEPCK Transcription: Nuclear Receptor-Mediated Downregulation of Hepatic Gluconeogenesis in Mice and a Human Hepatocyte Cell Line. PLoS ONE.

[21] Weinstock P.H., Bisgaier C.L., Hayek T., Aalto-Setala K., Sehayek E., Wu L., Sheiffele P., Merkel M., Essenburg A.D., Breslow J.L. (1997). Decreased HDL Cholesterol Levels but Normal Lipid Absorption, Growth, and Feeding Behavior in Apolipoprotein A-IV Knockout Mice. J. Lipid Res..

[22] Parlee S.D., Lentz S.I., Mori H., MacDougald O.A. (2014). Quantifying Size and Number of Adipocytes in Adipose Tissue. Methods Enzymol..

[23] Matthews D.R., Hosker J.P., Rudenski A.S., Naylor B.A., Treacher D.F., Turner R.C. (1985). Homeostasis Model Assessment: Insulin Resistance and Beta-Cell Function from Fasting Plasma Glucose and Insulin Concentrations in Man. Diabetologia.

[24] Tritos N.A., Mantzoros C.S. (1997). Leptin: Its Role in Obesity and Beyond. Diabetologia.

[25] Li S., Shin H.J., Ding E.L., van Dam R.M. (2009). Adiponectin Levels and Risk of Type 2 Diabetes: A Systematic Review and Meta-Analysis. JAMA.

[26] Franssen R., Monajemi H., Stroes E.S.G., Kastelein J.J.P. (2011). Obesity and Dyslipidemia. Med. Clin. N. Am..

[27] Abranches M.V., Oliveira F.C., Conceicao L.L., Peluzio M.D. (2015). Obesity and Diabetes: The Link between Adipose Tissue Dysfunction and Glucose Homeostasis. Nutr. Res. Rev..

[28] Davis B.H., Poon A.F.Y., Whitlock M.C. (2009). Compensatory Mutations Are Repeatable and Clustered within Proteins. Proc. Biol. Sci..

[29] Berglund E.D., Li C.Y., Poffenberger G., Ayala J.E., Fueger P.T., Willis S.E., Jewell M.M., Powers A.C., Wasserman D.H. (2008). Glucose Metabolism In Vivo in Four Commonly Used Inbred Mouse Strains. Diabetes.

[30] Romieu I., Dossus L., Barquera S., Blottière H.M., Franks P.W., Gunter M., Hwalla N., Hursting S.D., Leitzmann M., Margetts B. (2017). Energy Balance and Obesity: What Are the Main Drivers?. Cancer Causes Control.

[31] Brown L.M., Clegg D.J. (2010). Central Effects of Estradiol in the Regulation of Food Intake, Body Weight, and Adiposity. J. Steroid Biochem. Mol. Biol..

[32] Eckel L.A. (2011). The Ovarian Hormone Estradiol Plays a Crucial Role in the Control of Food Intake in Females. Physiol. Behav..

[33] Musatov S., Chen W., Pfaff D.W., Mobbs C.V., Yang X.-J., Clegg D.J., Kaplitt M.G., Ogawa S. (2007). Silencing of Estrogen Receptor Alpha in the Ventromedial Nucleus of Hypothalamus Leads to Metabolic Syndrome. Proc. Natl. Acad. Sci. USA.

[34] Hong J., Stubbins R.E., Smith R.R., Harvey A.E., Núñez N.P. (2009). Differential Susceptibility to Obesity between Male, Female and Ovariectomized Female Mice. Nutr. J..

[35] Cooke P.S., Naaz A. (2004). Role of Estrogens in Adipocyte Development and Function. Exp. Biol. Med..

[36] Wade G.N., Gray J.M., Bartness T.J. (1985). Gonadal Influences on Adiposity. Int. J. Obes..

[37] Shen L., Wang D.Q., Lo C.M., Tso P., Davidson W.S., Woods S.C., Liu M. (2010). Estradiol Increases the Anorectic Effect of Central Apolipoprotein A-IV. Endocrinology.

[38] Jia M., Dahlman-Wright K., Gustafsson J.-Å. (2015). Estrogen Receptor Alpha and Beta in Health and Disease. Best Pract. Res. Clin. Endocrinol. Metab..

[39] Pence S., Zhu Q., Binne E., Liu M., Shi H., Lo C.C. (2019). Reduced Diet-Induced Thermogenesis in Apolipoprotein A-IV Deficient Mice. Int. J. Mol. Sci..

[40] Thiriet M. (2019). Vasculopathies: Behavioral, Chemical, Environmental, and Genetic Factors.

[41] de Ferranti S., Mozaffarian D. (2008). The Perfect Storm: Obesity, Adipocyte Dysfunction, and Metabolic Consequences. Clin. Chem..

[42] Roberts R., Hodson L., Dennis A.L., Neville M.J., Humphreys S.M., Harnden K.E., Micklem K.J., Frayn K.N. (2009). Markers of de Novo Lipogenesis in Adipose Tissue: Associations with Small Adipocytes and Insulin Sensitivity in Humans. Diabetologia.

[43] Reue K., Purcell-Huynh D.A., Leete T.H., Doolittle M.H., Durstenfeld A., Lusis A.J. (1993). Genetic Variation in Mouse Apolipoprotein A-IV Expression Is Determined Pre- and Post-Transcriptionally. J. Lipid Res..

[44] Reue K., Leete T.H. (1991). Genetic Variation in Mouse Apolipoprotein A-IV Due to Insertion and Deletion in a Region of Tandem Repeats. J. Biol. Chem..

[45] Montgomery M.K., Hallahan N.L., Brown S.H., Liu M., Mitchell T.W., Cooney G.J., Turner N. (2013). Mouse Strain-Dependent Variation in Obesity and Glucose Homeostasis in Response to High-Fat Feeding. Diabetologia.

[46] Weinberg R.B. (2002). Apolipoprotein A-IV Polymorphisms and Diet-Gene Interactions. Curr. Opin. Lipidol..

