# Supplementary material for: Deficiency of apoA-IV in Female 129X1/SvJ Mice Leads to Diet-Induced Obesity, Insulin Resistance, and Decreased Energy Expenditure
Source: Nutrients. 2023 Nov 2;15(21):4655. doi: 10.3390/nu15214655 (PMC10650794; doi:10.3390/nu15214655)
Supplement: Supplementary file 1 [file nutrients-15-04655-s001.zip › nutrients-2670545-supplementary.pdf]

nutrients

Supplementary Materials

Deficiency of apoA-IV in Female 129X1/SvJ Mice Leads to Diet-Induced Obesity, Insulin Resistance, and Decreased Energy Expenditure

Jie Qu [1], Dong Wu [2], Chih-Wei Ko [3], Qi Zhu [4], Min Liu [4] and Patrick Tso [4,*]

Supplementary Figures:

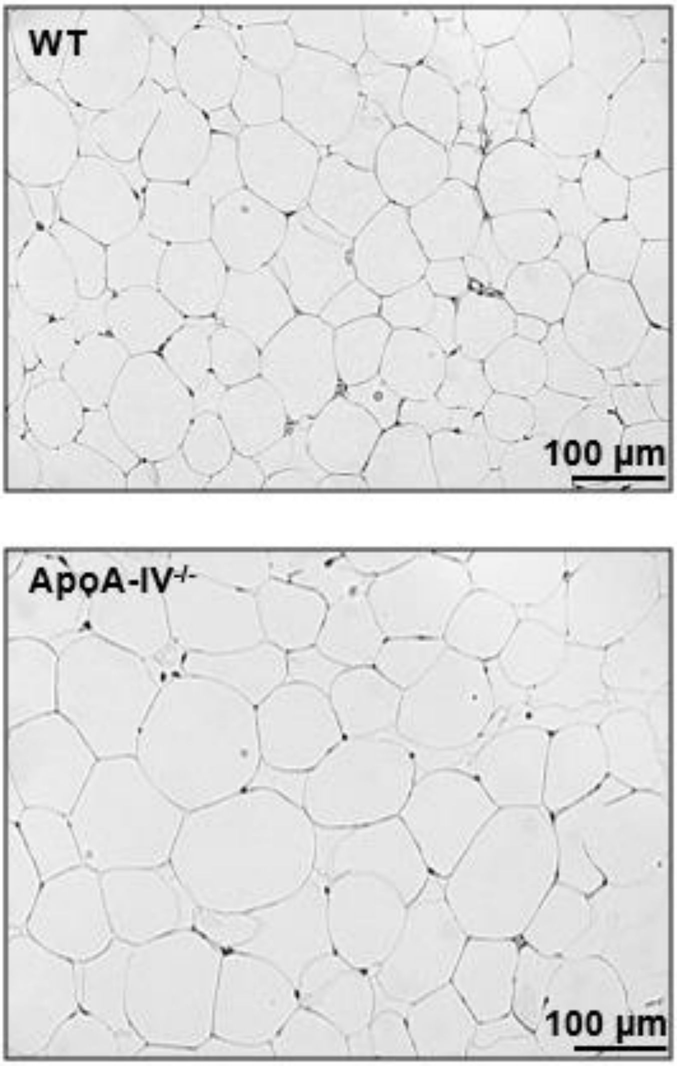

Figure S1. Representative sections of periovarian fat pads with H&E staining. Scale bar: 100 μm.

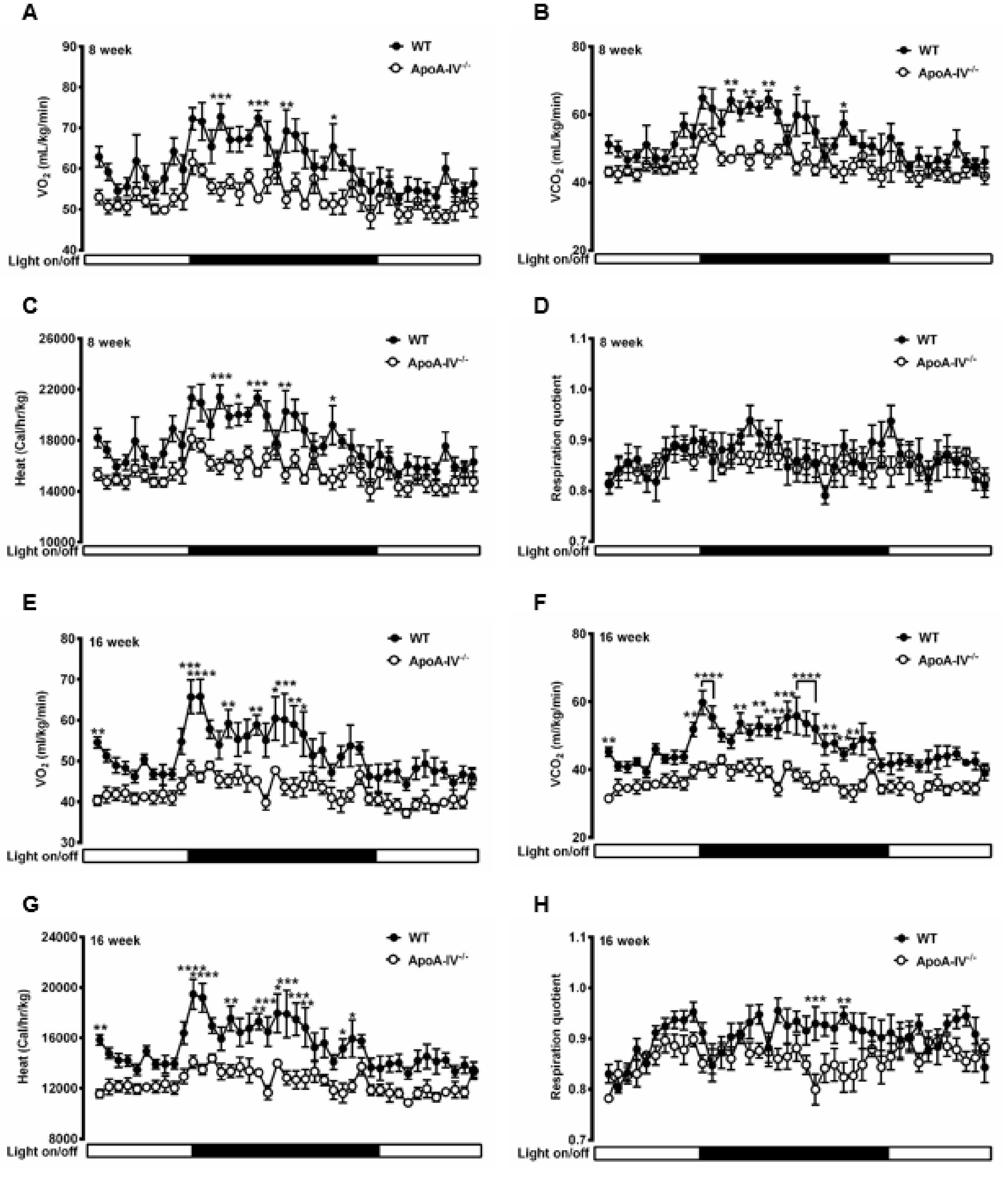

Supplemental Figure 2.

Figure S2. Indirect calorimetry measurement of WT and apoA-IV$^{-/-}$ mice at week 8 and week 16 of HFD. 24-hour measurements in oxygen consumption (A), carbon dioxide consumption (B), heat production (C), and respiration quotient (D) on Week 8 of HFD. 24-hour measurements in oxygen consumption (E), carbon dioxide consumption (F), heat production (G), and respiration quotient (H) on eek 16 of HFD. Data are presented as mean ± SEM (n=6) and analyzed statistically by regular two-way ANOVA. Significant differences relative to the WT group ($^{*}P < 0.05$; $^{**}P < 0.01$; $^{***}P < 0.001$; $^{****}P < 0.0001$).

Supplementary Table

Table S1. Tissue weight (g) of different fat depots.

Tissue	WT mice	ApoA-IV$^{-/-}$ mice
Periovarian fat	0.807 ± 0.081 g	1.840 ± 0.205 g
Subcutaneous fat	0.364 ± 0.022 g	0.752 ± 0.122 g
Retroperitoneal fat	0.179 ± 0.019 g	0.386 ± 0.042 g
Brown adipose tissue	0.090 ± 0.005 g	0.171 ± 0.008 g

Data are presented as mean ± SEM (n = 6).
